# Progress in Aptamer Research and Future Applications

**DOI:** 10.1002/open.202400463

**Published:** 2025-02-03

**Authors:** Song Liu, Xiaolu Li, Huyang Gao, Jing Chen, Hongfeng Jiang

**Affiliations:** ^1^ Beijing Anzhen Hospital Capital Medical University Experimental Research Center Beijing Institute of Heart Lung and Blood Vessel Disease Beijing China; ^2^ Guangxi Medical University Life Sciences Institute Nanning China

**Keywords:** Antibody, Aptamer, Aptamer characterization, Small molecule, Systematic evolution of ligands by exponential enrichment (SELEX)

## Abstract

Aptamers are short, single‐stranded DNA, RNA or synthetic XNA molecules that bind to target molecules with high specificity and affinity. These intrinsically structured RNA or DNA oligonucleotides are not only substitutes for antibodies, but also show great potential for applications in diagnostics, specific drug delivery, and treatment of certain diseases. While the process of aptamer identification and its core functional mechanism known as systematic evolution of exponentially enriched ligands (SELEX), SELEX involves a number of single processes, each contributing to the success or failure of aptamer generation. Today, aptamers are widely used to facilitate basic research discoveries and clinical diagnostics. In addition, aptamers play a promising role as clinical diagnostic and therapeutic agents. This review provides recent advances in this rapidly growing field of research, with special emphasis on aptamer generation and screening, small molecule aptamers, the development of aptamer applications, and applications in clinical medicine. And it also discusses the problems that still exist today with aptamers.

## Graphical abstract

This paper mainly describes the screening method of aptamer, the screening difficulties and improvement methods of small molecule aptamer, and the application and treatment of aptamer. It explores the latest progress and application of aptamers, and summarises them, thus indicating some problems that still exist in this field.

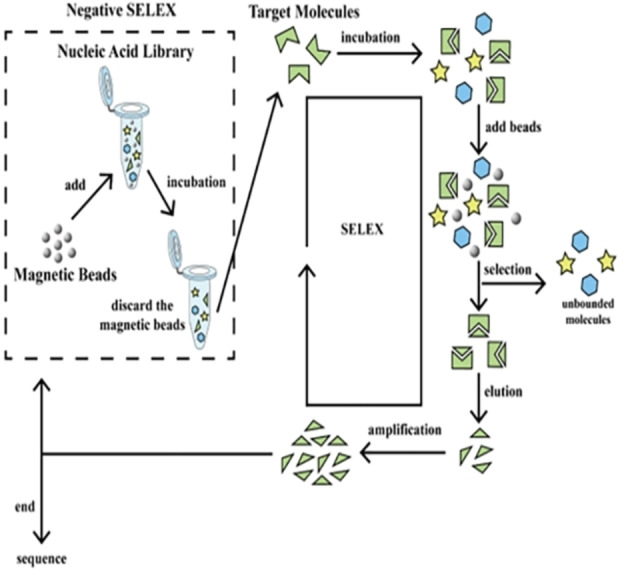

## 1 Introduction

Nucleic acids, such as DNA and RNA, were long thought to primarily function as genetic material. However, with deeper understanding and research, it is now recognised that nucleic acids not only carry genetic information^[1]^ but also perform various biological functions, such as catalytic and transcriptional regulation.^[2]^ The selection and application of aptamers is also an important area of research regarding the multifunctionality of nucleic acids.^[3]^ Structurally, an aptamer is a short oligonucleotide sequence (ssDNA or RNA) typically composed of 20–60 nucleotides. It not only exhibits extremely high specificity but also has a strong affinity for its target molecule.^[4]^ Therefore, aptamers are typically used as recognition elements in clinical diagnostics and therapeutics. Aptamer selection is primarily conducted through Systematic Evolution of Ligands by Exponential enrichment (SELEX). This method was first reported by Tuerk and Gold,^[5]^ as well as Ellington and Szostak^[6]^ in 1990. Numerous aptamer‐targeting proteins and small molecules have since been developed.^[7]^

Aptamers and antibodies are highly similar in function as they both exhibit extremely high specificity and affinity for binding to their target molecules. Over 2,000 aptamers have been generated in recent decades.^[8]^ Aptamers have certain advantages over antibodies. They exhibit strong inertness towards non‐target cells, which means that specific aptamers have extremely high specificity for their target molecules.^[2]^ Furthermore, compared to antibodies, aptamers are smaller, which facilitates their ability to penetrate deeper into tissues and reach intracellular targets^[9]^ Additionally, they exhibit shorter selection cycles, minimal batch‐to‐batch variability, and ease of large‐scale production. These benefits make aptamers strong candidates to replace antibodies in therapeutic applications.^[10]^ See Table 1 for a detailed comparison of the advantages and disadvantages of antibodies and aptamers.

**Table 1:** Comparison of the advantages and disadvantages of aptamers and antibodies.

	Aptamer	Antibodies	Ref.
Molecular weight	5–30 kDa	150–180 kDa	[2,11]
Progress	SELEX	B‐cell immune response	
Structures	Various structures: G‐quadruplexes and hairpin	Various structures: G‐quadruplexes and hairpin	
Generation time	Several weeks	Months	
Batches variations	Low	High	
Immunogenicity	None or extremely low	High	
Modifiability	Easy to modify without reducing affinity	Frequent modification leads to decreased activity	
Nuclease degradation	Sensitive	Insensitive	
Stability	Stabilize	Easily affected by environment, temperature, and pH	
Targets	Ions, small molecules, macromolecules, cells, animals	Substances that can generate immune responses	
Shelf life	Long	Short	
Renal filtration	Fast	Slow	
Cost	Low	High	

Despite the many advantages of aptamers over antibodies, there are still some challenges that need to be addressed, including the design of specific libraries, the toxicity and stability of aptamers, and the optimisation of selection conditions.^[11a]^ This is mainly due to the fact that SELEX technology involves a very stringent selection process, which results in a relatively low success rate.^[11b]^ Additionally, aptamers are developed in vitro with the ultimate aim of clinical application. However, it remains to be seen whether aptamers created in vitro can function effectively in the human body.^[12]^ In this review, we discuss the latest advancements in aptamer selection technologies, challenges faced in the selection of small‐molecule aptamers, and their prospects for clinical diagnostics and therapeutics.

## 2 Generation and Selection of Aptamers

Generation and traditional SELEX: Aptamer selection is performed using SELEX technology.^[13]^ SELEX primarily involves several steps: library selection, incubation, binding, washing, and amplification.^[14]^ Before selecting the target molecule, an oligonucleotide library containing approximately 1012 1016 sequences is synthesised, with variable sequences in the central region and fixed sequences at the ends for primer binding and PCR amplification. The target molecules are incubated with this library. After incubation, bound sequences are separated from unbound ones using appropriate methods. The bound sequences are then amplified using PCR.^5^, ^15^ The resulting products serve as a pool for the next round of selection. With each cycle, the pool becomes increasingly enriched with specific sequences. These enriched sequences are then sequenced, and their functionality and binding affinity are validated and assessed.^[16]^ Generally, obtaining an aptamer for a specific molecule can take several months, with a relatively low success rate. Therefore, obtaining high‐quality aptamers for target molecules remains challenging.^[11b]^

Negative‐SELEX: To obtain aptamers for a specific target substance, it is usually necessary to immobilise the target molecule on solid‐phase supports, often using magnetic beads. However, nonspecific binding to the surface of magnetic beads can lead to false positives, resulting in deviations in the final results. To minimise these errors, Ellington and Szostak introduced a new method called Negative‐SELEX in 1992.^[17]^ Before incubating the target with the library, magnetic beads are incubated with the library alone to induce nonspecific binding. The beads are then discarded. Aptamers obtained using this method exhibit approximately 10 times higher affinity than those obtained without negative selection.^[12]^

Toggle‐SELEX: This method involves the use of multiple positive targets to screen all molecules that bind to a target, allowing for dynamic changes in the target conditions during the selection process. It is commonly used to select aptamers that target tumour cells.^[18]^ Because tumour cell surface epitopes constantly mutate, leading to drug resistance, it is necessary to use this method to ensure that the drug treatment remains effective despite these mutations. This method was used to select aptamers as anticoagulants.^[19]^ It has also been used to obtain aptamers against various gram‐negative bacteria.^[20]^

Capture SELEX: This is a method for immobilising the library.^[21]^ Typically used to isolate small‐molecule aptamers,^[22]^ this method involves immobilising a library on a solid‐phase support. Heating and cooling are used to facilitate the binding of the library to oligonucleotides. Once the complex is formed, streptavidin‐coated magnetic beads are added to bind the biotinylated oligonucleotide sequences, creating a magnetic bead‐oligonucleotide complex. Specific aptamers are subsequently obtained through washing and elution.^[23]^ In 2012, Regina et al. successfully selected aptamers for the aminoglycoside antibiotic, kanamycin A.^[24]^ Additionally, Georges et al. (2017) successfully selected aptamers for penicillin G.^[25]^

Cell‐SELEX: This method primarily utilises live cells as targets to identify aptamers that selectively bind to cell surface receptors or recognise specific cell populations.^[26]^ In addition, compared to in vitro SELEX, Cell‐SELEX is based on natural structures on cell surfaces, making it more applicable in clinical settings compared to artificially designed conformations.^[27]^ In addition, Cell‐SELEX can identify aptamers that target a broad range of locations, which is important for understanding and addressing clinical diseases. In 2003, Dion et al. successfully selected aptamers for tenascin‐C using the glioblastoma‐derived U251 cell line,^[28]^ as well as aptamers for glioma cells.^[29]^

Capillary Electrophoresis ‐SELEX(CE‐SELEX): CE‐SELEX is a technique that separates ions based on their electrophoretic mobility. Compared to traditional SELEX, it requires fewer selection rounds (typically only to 1–4 cycles) to obtain high‐affinity aptamers.^12^, ^30^ CE‐SELEX separates bound DNA molecules from unbound ones in solution and then uses solid‐phase supports and washes to eliminate linkers. However, this method is expensive and is limited to the selection of high‐molecular‐weight targets. Many aptamers have been successfully selected using CE‐SELEX, including those for alpha‐fetoprotein^[31]^ and GPC3.^[32]^ Notably, GPC3 has been identified as a tumour biomarker for the early diagnosis of hepatocellular carcinoma.

Microfluidic SELEX (M‐SELEX): In traditional SELEX, the process of multiple rounds of enrichment inevitably leads to errors such as sequence loss, increased nonspecific amplification, and contamination during PCR. M‐SELEX was designed to address these issues.^[33]^ M‐SELEX is an automated technique that rapidly screens aptamers with high specificity and affinity at the microscale level. The most notable feature is the improved efficiency of micromagnetic separation devices.^[34]^ Studies have shown that reducing the number of targets can quickly identify a few sequences within the aptamer pool to increase the specificity of the selection process.^[13]^

High‐throughput sequencing SELEX: HTS‐SELEX is an improvement over the classical SELEX method.^[5]^ This technique allows sequencing in any round of the selection process, which helps prevent contamination or loss of selected sequences due to operational or environmental issues, ensuring that the desired sequences are always present.^[12]^ HTS‐SELEX can quantify the specificity of target molecules with high precision,^[35]^ and is frequently used to analyse the binding specificity of RNA.^[36]^ In 2015, Daniel et al. successfully selected and enriched 2′‐fluoropyrimidine‐modified RNA aptamers specific to plasminogen activator inhibitor‐1 (PAI‐1) using high‐throughput sequencing technology.^[37]^ Current research suggests that integrating microfluidics with HTS technology can greatly advance the study of aptamer kinetics.^[38]^

In vivo SELEX: Conventional SELEX methods are typically performed in vitro, with targets often synthesised artificially. To address the issue of aptamers selected in vitro that do not function effectively in vivo, researchers have developed in vivo SELEX. This method is commonly used in drug delivery systems and allows for the generation of aptamers that can penetrate tissues directly in animal models of target diseases. In 2018, Chen et al. used this method to identify bone‐targeting aptamers in a mouse model with phosphatidylserine‐binding proteins (PBs).^[39]^ In 2010, Mi et al. first attempted to obtain aptamers by injecting them into live tumours.^[40]^ Furthermore, in 2020, Cesarini et al. developed a targeted drug delivery system capable of crossing the blood–brain barrier through in vivo targeted therapies.^[41]^ Extensive experiments have demonstrated that the generation of aptamers in vivo is feasible.

Micro column SELEX: Microcolumn SELEX is a highly efficient aptamer selection method that utilizes microcolumn technology to achieve high‐throughput screening, significantly enhancing screening efficiency. This technique has been extensively applied in biomedical research, food safety, and other fields. Despite its relatively high cost and technical complexity, Microcolumn SELEX remains valuable due to its ability to select aptamers with high affinity and specificity.^[42]^

Atomic force microscopy SELEX: Atomic force microscopy (AFM) has demonstrated unique advantages in SELEEX (Systematic Evolution of Ligands by an Exponential enrichment) technology, which dynamically detects the adhesion force between the sample surface and the cantilever, and effectively screens for high‐affinity aptamers. For example, Miyachi et al. successfully screened high‐affinity thrombin aptamers by AFM‐SELEX.^[43]^

Size exclusion chromatography SELEX: Size exclusion chromatography (SEC) is used in SELEEX (Systematic Evolution of Exponentially Enriched Ligands) technique to separate molecules of different sizes, effectively improving the efficiency and accuracy of aptamer screening. For example, in the study of Varcheha et al, a highly specific aptamer for human plasma coagulation factor VIII was successfully screened using SEC‐SELEX (Table 2).^[44]^

**Table 2:** Types of SELEX techniques.

Method	Characteristic	Disadvantages	Application	Ref.
Negative‐SELEX	Improve specificity Enhance affinity Improve aptamer performance	More optimisation is needed High resource consumption Increased experimental complexity	Detection of cancer biomarkers Developing antiviral adaptors Development of Specific Drug Delivery Systems	[45] [46] [47]
Toggle‐SELEX	Multifunctional applications Adapt to complex environments Reduce nonspecific binding	High technical requirements Long experimental time	Protein detection in biosensors	[48]
Capture SELEX	Introducing capture steps Enhanced selectivity Suitable for multiple goals	Introducing capture steps may increase complexity High requirements for solid‐phase materials Possible selection bias	Virus detection Development of biosensors Cancer diagnosis and treatment	[49] [50] [51]
Cell‐SELEX	Identifying natural conformational targets Identifying unknown targets Simplified target recognition	Complexity and time cost Differences in stability of cell lines The complexity of the library is high	Cancer diagnosis and treatment Cell labelling and imaging regenerative medicine	[52] [53] [54]
CE‐SELEX	High sensitivity High throughput Automation and efficiency Wide range	High technical requirements Complex data analysis High purity requirements for samples	Development of biosensors Research on diagnostic tools	[55] [56]
M‐SELEX	High‐throughput screening Low sample consumption	High Device Dependency Complex chip design	Drug screening and design	[57]
HTS‐SELEX	High‐throughput screening Comprehensiveness Powerful data analysis capabilities	Expensive expenses High professional and technical requirements Difficulty in data processing	Drug discovery and development Disease diagnosis	[58] [59]
In vivo SELEX	Improved biological relevance Wider application potential Used for disease diagnosis and treatment	The screening process is more complex and time‐consuming More technical difficulty High cost	Targeted tumour therapy Pathogen detection and treatment Drug delivery	[41] [60] [61]
Microcolumn SELEX	High‐throughput screening Resource conservation Wide range of applications	High costs technically complex Long screening cycles	Disease diagnosis and treatment Food safety testing environmental monitoring	[62]
Atomic force microscopy SELEX	High sensitivity and resolution Real‐time dynamic monitoring	High cost technically complex Long screening cycles	biomedical research Food safety testing environmental monitoring	[43]
Size exclusion chromatography SELEX	High purity screening Maintains aptamer activity	High sample requirements High cost	Disease diagnosis and treatment Drug discovery and development biosensor	[44]

## 3 Small Molecule Aptamers and Sensors

With advancements in medicine and improvements in safety awareness, the demand for biosensors is increasing. Small‐molecule aptamers play an increasingly important role in the field of biosafety because of their low detection thresholds and ability to rapidly detect low‐quality compounds, such as environmental toxins, antibiotics, drugs, and heavy metals. Additionally, precise disease diagnosis requires the detection of disease‐related proteins, metabolic markers, and other compounds,^[63]^ thus increasing the demand for precise small‐molecule detection. However, despite the growing demand, biosensors for these trace compounds have not been studied as extensively as those for protein targets. The primary challenge is the difficulty in screening small‐molecule aptamers.^[64]^ Currently, the primary methods for detecting small molecules rely on antibodies or enzymes for binding or reactions to detect the presence of small molecules. However, these methods have limitations, often being limited to specific targets, particularly non‐immunogenic substances. Additionally, owing to the structural diversity of small molecules, their specificity often tends to be low.

Small‐molecule aptamers have several advantages over traditional biosensors. Aptamers are structurally flexible, serving as highly specific receptors. They can be selected for a wide range of target molecules, including toxic and non‐immunogenic compounds. Despite the structural variability of small molecules, aptamers exhibit high specificity for structurally similar compounds.^[65]^ These characteristics indicate their potential for the development of novel and unique nucleic acid aptamer detection technologies.^[66]^ The structural flexibility of aptamers is also reflected in their complex three‐dimensional conformations, which allow them to form pocket‐like structures that bind to their targets. This theoretically enables them to interact with a wide range of target molecules.

## 4 Challenges in Screening Small‐Molecule Aptamers

Small‐molecule aptamer screening is one of the most challenging aspects of aptamer selection, primarily because of the characteristics of small molecules (Table 3).

**Table 3:** Differences in the physicochemical properties of small molecules and proteins.

Properties	Small molecules	Proteins
Molecular weight	<500 Da	>10 kDa
Structural complexity	Structural simplicity, with fewer atoms	Complex structure with multiple levels of organisation
Solubility	Variable solubility due to structural differences	Mostly hydrophilic
Synthesis	Simple to synthesise: can be obtained through chemical synthesis or biosynthesis methods	Ribosomal synthesis: achieved through transcription and translation from DNA‐encoded sequences
Distribution	Small size: allows for distribution within the organism and the ability to cross biological membranes, such as the blood–brain barrier	Larger Size: distribution is restricted to specific biological environments, requiring special mechanisms to cross membranes or move between cells
Target binding epitope	Fewer	More
Conjugatable regions	Limited, but with high chemical diversity, requiring specialised chemical components for conjugation	Abundant, with minimal impact on aptamers

**Figure 1:**
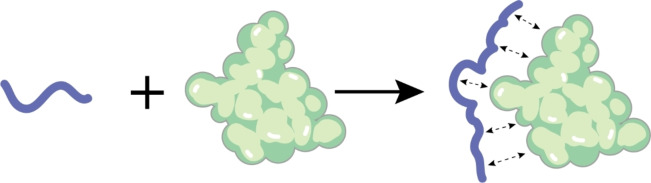
Aptamers bind to their targets through conformational interactions.

Compared to large‐molecule targets such as proteins, small molecules have smaller molecular weights, smaller surface areas, and lower structural complexity. This not only results in fewer surface binding sites but also limits the strength of possible interactions between functional groups, ligands, and aptamers, ultimately leading to reduced affinity.^[67]^ The functional groups of small molecules are more diverse, necessitating specialised chemical components for immobilisation on the target. Additionally, target immobilisation may generate novel epitopes required for aptamer interactions. However, since aptamers rely on hydrogen bonding and electrostatic interactions,^[68]^ π–π stacking, van der Waals forces, and conformational adaptability for binding (Figure 1), the limited number of functional groups not only affects the specificity of the screening process but may also result in reduced affinity between the aptamer and the target molecule.^[69]^

While no absolute procedure can be universally applied for the selection of small‐molecule aptamers, it is possible to predict the difficulty of aptamer selection based on the physicochemical properties of the target molecule.^[70]^ Generally, aptamers are easier to isolate from larger target molecules because they have more epitopes available for binding. In addition, because nucleic acid aptamers carry a negative charge, it is easier to obtain high‐affinity binders for both positively and negatively charged targets.

### 4.1 Methods for Isolating Small‐Molecule Aptamers

#### 4.1.1 Methods to Enhance the Specificity of Small‐Molecule Aptamer Isolation

The most crucial step in the aptamer selection process is the immobilisation of the target molecule on magnetic beads, known as target immobilisation. However, this method has a significant drawback: the magnetic beads themselves may exhibit nonspecific binding, which can reduce the specificity of the final aptamer product. Negative‐SELEX effectively addresses this issue.^[17]^ Firstly, the library is incubated with unmodified magnetic beads to promote nonspecific binding to the beads. The beads are discarded, and the remaining library is incubated with target‐immobilised beads for positive selection. Counter‐SELEX is similar to Negative‐SELEX in that both methods aim to remove nonspecific binding sequences from the library, thereby ensuring that isolated aptamers have high specificity.^[71]^ Negative‐SELEX primarily enhances specificity by addressing nonspecific binding to magnetic beads, whereas counter‐SELEX improves specificity by introducing counter targets into the library. In counter‐SELEX, counter targets and their binding sequences are discarded to ensure higher specificity of the final aptamers. For example, Hadi et al. (2021) incorporated counter‐SELEX into cell‐SELEX to address the issue of low specificity in the selection process. This improved method successfully overcame the limitations of selecting aptamers targeting aspartate β‐hydroxylase (ASPH) as tumour markers.^[72]^ In addition to negative selection, structurally related compounds such as highly similar drugs are sometimes involved. SELEX can be used to isolate aptamers specific to groups of highly similar compounds, a technique named Toggle‐SELEX by Sullenger et al. in 2001.^[19]^ This method employs multiple positive targets to screen for all molecules that bind to the target. Due to its unique separation characteristics, toggle‐SELEX is often used to isolate tumour cells. It has also been demonstrated to be an effective method for isolating aptamers that cross‐react with proteins and cell targets.^[73]^

#### 4.1.2 Methods to Enhance the Affinity of Small‐molecule Aptamers

Compared to the antibody production process, SELEX technology offers the advantage of precise control over experimental conditions, allowing selective enrichment of the desired aptamers. For example, lower stringency can be used to screen and enrich aptamers with lower affinity, whereas higher stringency can be used to select aptamers with the highest affinity.^[74]^ Stringency can be adjusted by varying conditions, such as the concentration of the library and target molecules, incubation time, temperature, and pH.^74^, ^75^ In practice, while high stringency is often used to select high‐affinity aptamers, this approach may risk losing some sequences. Therefore, lower stringency is typically used during the initial rounds of selection to retain as many binding sequences as possible. Stringency is gradually increased in subsequent rounds to ensure the selection of aptamers with the highest affinity.^[76]^

However, owing to the stringent requirements of SELEX technology, high‐affinity aptamers may not always be obtained during experiments. Therefore, the affinity of aptamers can be increased through various adjustments.

Structural modifications: The more structurally stable the aptamer is with constant affinity, the more likely it is to maintain conformation in complex biological environments and thus bind effectively to target molecules. Locked nucleic acids (LNAs) are a type of modified nucleic acids that stabilise the aptamer structure by using a methylene bridge between the 2′‐O and 4′‐C atoms of the ribose, thus enhancing nucleic acid stability. Although LNAs are synthetic, they can still be paired with natural nucleotides. Notably, the incorporation of LNA and nucleotides into DNA and RNA can increase their melting temperatures.^[77]^ Therefore, LNAs are often used to modify double‐stranded structures of nucleic acid aptamers.

Library modifications: Another method to enhance the affinity of aptamers for their targets is to increase the number of interactions between the aptamer and the target. This can be achieved by simulating the addition of functional groups to amino acid side chains and potentially increasing the chemical diversity, which can enhance the binding affinity of the aptamers.^[78]^ However, because SELEX with modified libraries has a high selectivity for nucleic acids, methods involving modified libraries based on SELEX face significant challenges. Notably, Vaught et al. developed modified oligonucleotides suitable for SELEX enzymes.^[79]^ Additionally, Gold et al. tested modified oligonucleotides using 13 human proteins.^[80]^

### 4.2 Characterisation of Small‐molecule Aptamers

Before aptamers can be applied to real‐world problems, their affinity, specificity, potential side effects, and resistance must be thoroughly understood. These characteristics are closely related to the ultimate applications of aptamers. Studying the interactions between aptamers and small molecules requires specialised equipment and a deep understanding of thermodynamics.

**Figure 2:**
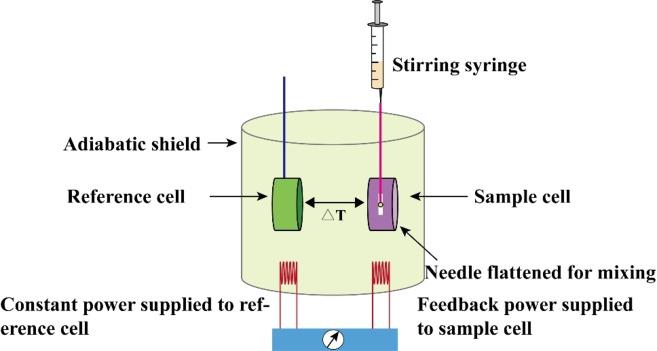
ITC Working Principle Diagram.

Isothermal titration calorimetry (ITC): At the beginning of the 21st century, ITC was used to study the interactions between small molecules and aptamers.^[68]^ Since then, this method has been extensively used in other applications.^[81]^ Notably, ITC has been widely used as a label‐free thermodynamic method to determine the affinity between nucleic acid aptamers and target molecules by measuring the heat released during interactions (Figure 2).^[82]^ For example, Tran et al. (2022) used ITC to determine the thermodynamic and kinetic interactions between DNA aptamers and tetracycline.^[83]^

**Figure 3:**
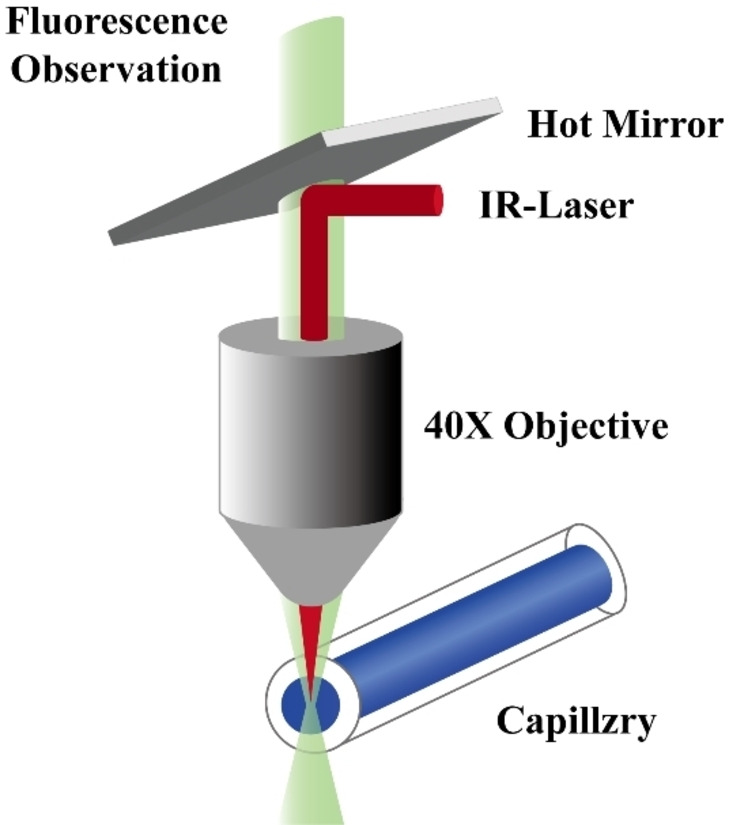
MST Working Principle Diagram.

Microscale thermophoresis (MST): MST is an innovative method increasingly used to study the dissociation constant (K_D) of small‐molecule nucleic acid aptamers.^[84]^ Moreover, this method is also a thermodynamic technique,^[85]^ involving the subjection of molecules to an temperature gradient via thermophoresis. The movement of molecules along this gradient can lead to molecular loss.^[86]^ This method is commonly used to determine the affinity constants between molecules (Figure 3).^[87]^ Rangel et al. indicated that the greatest advantage of MST is its ability to measure the K_D value in complex samples, observing that it can be used to detect aflatoxin A aptamers in human serum.^[88]^

**Figure 4:**
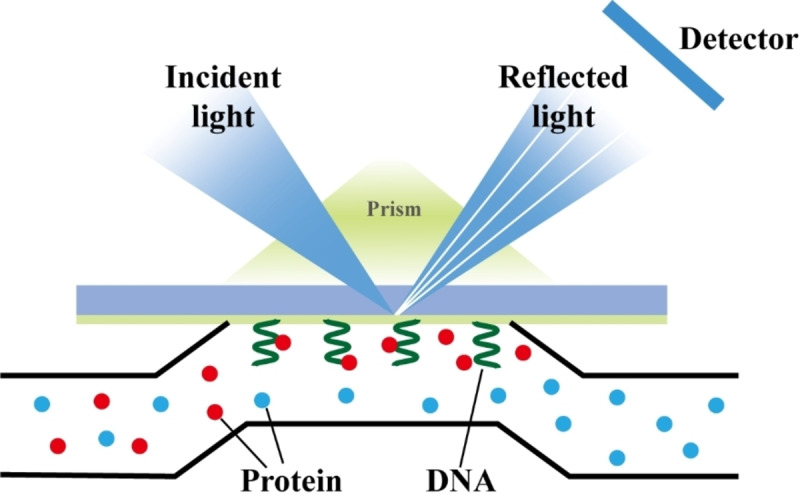
SPR Working Principle Diagram.

Surface plasmon resonance (SPR): SPR is a standard method for measuring the binding parameters of nucleic acid aptamers.^[89]^ This method offers various advantages, such as high throughput, real‐time monitoring, and label‐free analysis.^[90]^ This method can be used to determine the affinity and kinetic properties of nucleic acid aptamers for target molecules. The traditional approach involves immobilising a molecule or nucleic acid aptamer of interest on a sensor chip surface, followed by detection in solutions of varying concentrations. To measure binding affinity, changes in the refractive index caused by the formation of the aptamer–target complex are recorded (Figure 4).^[91]^ However, a major limitation of SPR technology is that it requires the immobilisation of the ligand on the sensor chip surface. This immobilisation can affect the binding ability of the ligand.^[92]^ In 2014, Chang et al. developed a universal characterisation method that effectively avoids target immobilisation, potentially hindering aptamer binding.^[93]^

In summary, selecting small‐molecule aptamers remains challenging but is a highly active research area with broad applications across various fields.

## 5 Applications of Aptamers

Aptamers are now widely used in various fields because of their ability to specifically recognise and bind to target molecules.^[94]^ Consequently, their applications in clinical diagnostics and treatment are becoming more frequent, including applications in areas such as biosensors, pathogen detection, and cancer diagnosis.

Applications in biomedical diagnostics: Because of their strong affinity and specificity, aptamers have significant potential in clinical diagnostics and related fields. In addition, aptamers are easily labelled. In 1999, Bruno et al. developed the first aptamer as a diagnostic tool. They used an aptamer to detect anthrax spores.^[95]^ In addition, aptamers have significant potential in precision medicine for both treatment and diagnosis. Modified aptamers can resist enzymatic degradation, thereby ensuring their integrity and utility. Thus, they play important roles in targeted therapies.^[96]^

Applications in antiviral therapy: Extensive research has been conducted on aptamer‐targeting viruses. In 1996, Burke et al.^[97]^ and in 2006, DeStefano et al.^[98]^ successfully developed aptamers for the human immunodeficiency virus that can effectively inhibit its activity. In 2004, Jeon et al.^[99]^ and in 2006, Gopinath et al.^[100]^ successfully screened aptamers for influenza virus. These aptamers effectively prevented the virus from invading the cells. Building on this, a nucleic acid aptamer was developed to distinguish between different strains of the same virus and applied for influenza virus genotyping.^[101]^ In 2002, Biroccio et al.^[102]^ and in 2014, Gao et al.^[103]^ successfully screened aptamers against hepatitis viruses. In 2021, Veesler, S. H et al. successfully developed the SARS‐CoV‐2 aptamer, which has shown great potential as a novel biorecognition tool for novel coronavirus detection and therapy. By specifically recognising viral proteins such as spiculin, the aptamer not only improves detection sensitivity, but also facilitates the development of novel antiviral therapeutics, providing an important tool in the fight against global pandemics.^[104]^ These aptamers have been shown to inhibit enzyme activity and suppress virus particle formation by blocking the interactions between proteins, RNA, and host proteins.

Applications in cancer detection: Currently, the most commonly used anticancer drugs are nonspecific and can cause varying degrees of damage to human health, leading to significant side effects.^[105]^ However, aptamers can be conjugated with other agents to serve as drug delivery systems, thereby reducing the damage to normal cells and enhancing therapeutic efficacy.^[106]^ Various tumour cell aptamers have been successfully screened using SELEX. For example, aptamers against the prostate‐specific membrane antigen have been identified^[107]^ Furthermore, aptamers targeting epithelial cell adhesion molecule 1^51^, ^108^ carcinoembryonic antigen (CEA)^[109]^ oestrogen receptor^[110]^ and protein tyrosine kinase 7^[111]^ have been developed. These aptamers serve as tumour markers that play crucial roles in cancer detection.

Applications in regenerative medicine: Aptamers show great potential in stem cell regenerative medicine. They can modify the extracellular matrix to provide a favourable environment for stem cell growth and differentiation, thus promoting tissue regeneration. With the advancement of SELEX technology, nucleic acid aptamers can now be used for classifying target cells. They are characterised by low production costs, strong resistance to enzymatic degradation, high purity, high efficiency, and minimal impact on cells. Currently, a large number of aptamers with high specificity and affinity for various stem cells have been identified. For example, in 2021, de Melo et al. successfully screened aptamers for human adipose tissue‐derived stem cells.^[111b]^ In 2020, Yao et al. successfully captured bone marrow mesenchymal stem cells (MSCs).^[112]^ In 2006, Guo et al.^[113]^ and in 2007, Schäfer et al.^[114]^ successfully isolated aptamers for adipose‐derived mesenchymal stem cells (aMSCs) from pig bone marrow.

Applications in biosensors: The core components of biosensors are receptors and transducers. Biological receptors typically comprise antibodies, cells, enzymes, and nucleic acids.^[115]^ Nucleic acid aptamers, which are biological receptors, typically recognise and bind to target biomolecules.^[116]^ Subsequently, the signal transducer interacts with the target substance and biological receptor to generate a detectable signal.^[117]^ Currently, most biosensors are based on antibody–antigen binding.^[118]^ Aptamers are promising new types of optical and electrical biosensors.^[119]^ In 2015, Chung et al. successfully conjugated aptamers with fluorescent nanoparticles (A‐FNPs) and detected Escherichia coli.^[120]^ Moreover, in 2015, Abbaspour et al. detected Staphylococcus aureus using an electrical sensor.^[121]^

## 6 Aptamers in Clinical Trials

Currently, aptamers targeting cancer, antimicrobial agents, anticoagulants, anti‐inflammatory drugs, AMD aptamers, and Aptamers not yet in clinical use are undergoing clinical trials. In this section, we focus on the development of these nucleic acid aptamers.

### 6.1 Anti‐Tumour Aptamers

Cancer has been a relentless adversary for centuries.^[122]^ However, effective treatments for many types of tumours are still lacking, primarily because of the absence of suitable molecular biomarkers and targets.^[123]^ In recent years, significant progress has been made in antibody‐based targeted therapies.^[124]^ However, owing to their high development costs, lengthy timeframes, and the potential to provoke strong immune responses that can affect drug efficacy, their use is limited.^[125]^ In contrast, nucleic acid aptamers are cost‐effective, easy to synthesise, and exhibit properties similar to those of antibodies.

#### 6.1.1 AS1411

Nucleolin is overexpressed in many cancers,^[53]^ and AS1411 significantly reduces the viability of cancer cells overexpressing nucleolin. Cheng et al. found that in gliomas, AS1411 inhibits nucleolin expression while increasing the expression of the tumour suppressor gene p53 and decreasing the expression of Bcl‐2 and Akt1. This indicated that AS1411 directly inhibited nucleolin.^[126]^ Although AS1411 is a promising tumour‐targeting drug, its exact mechanism of action remains unclear.^[127]^

#### 6.1.2 IDO‐APT

IDO1 is a heme‐containing dioxygenase that acts as a classical immune checkpoint and is thought to contribute to tumour immune escape. IDO1 is a very promising target for cancer immunotherapy and a number of chemical inhibitors of IDO1 have been developed including indoximod (1‐methyl‐D‐tryptophan, 1‐MT) and Yoon et al. showed that IDO1‐APT could effectively delay tumour growth and enhance the immune response by fractionating IDO1‐APT.^[128]^

### 6.2 Antimicrobial Aptamers

Antimicrobial aptamers have broad applications in the treatment of infections, including diagnosis, therapy, and prevention of bacterial infections. Compared to traditional antibiotics, aptamers are less likely to induce bacterial resistance and offer greater flexibility in drug design. For example, aptamers target S. aureus can bind to surface proteins such as staphylococcal protein A^[129]^ or cell wall components,^[130]^ inhibiting infection.^[131]^ Aptamers targeting E. coli have also been developed, including those specific to E. coli K88^[132]^ and E. coli NSM59 isolated from faecal samples.^[133]^

### 6.3 Anticoagulant Aptamers

Venous thrombus formation is a significant cause of morbidity and mortality worldwide,^[134]^ requiring anticoagulants and antiplatelet agents for treatment.^[135]^ Next, we introduce two aptamers related to anticoagulation.

#### 6.3.1 REG1

REG1, developed by Regado Biosciences (New Jersey, USA), is an IXa inhibitor based on nucleic acid aptamers.^[136]^ Factor IX plays a crucial role in the initiation and propagation of coagulation. Several drugs targeting Factor IX have been developed and have shown significant efficacy.^[137]^ Additionally, the risk‐to‐benefit ratio of bleeding versus anticoagulation with Factor IX inhibition is favourable.^[138]^ This indicates that Factor IX could be a promising target for the development of anticoagulants.

#### 6.3.2 ARC1779

ARC1779 is an aptamer that targets the VWF A1 domain.^[139]^ VWF interacts with platelets to rapidly form platelet thrombi, thereby preventing bleeding.^[140]^ However, in certain VWF‐mediated diseases, such as coronary artery syndrome,^[141]^ cerebral infarction,^[142]^ and thrombotic thrombocytopenic purpura,^[143]^ VWF levels are significantly elevated. Therefore, the inhibition of VWF activity is an effective strategy for treating VWF‐related diseases.

### 6.4 Anti‐Inflammatory Aptamers

Anti‐inflammatory aptamers are single‐stranded nucleic acids or peptide sequences with high specificity and affinity that can effectively inhibit inflammatory responses.

#### 6.4.1 Anti‐TNF‐α Aptamers

The TNF‐α pathway plays a key role in inflammatory diseases. During the injury phase, numerous neutrophils and macrophages are activated.^[144]^ These inflammatory cells secrete TNF‐α and other inflammatory factors. However, overactivation of this pathway can lead to an excessive release of inflammatory factors, resulting in cell death. In some diseases, TNF‐α levels are negatively correlated with survival outcomes.^144^, ^145^ TNF‐α acts as a “double‐edged sword” in the regulation of the immune system. Although optimal concentrations are crucial for the body's natural immune response to bacterial infections, overproduction can be detrimental.^[146]^

#### 6.4.2 Anti‐IL‐6 Aptamers

IL‐6 is an inflammatory mediator that plays a significant role in various inflammatory diseases. Rheumatoid arthritis (RA) is a common chronic inflammatory disease often accompanied by joint inflammation and damage,^[147]^ and is associated with IL‐6. While RA remains incurable, treatment strategies aim to slow its progression, with methotrexate being a common therapy. The IL‐6 receptor antibody tocilizumab is also used to block IL‐6 signalling.^[148]^ Lowering IL‐6 levels to normal is an effective approach in managing RA.

#### 6.4.3 VCAM‐1 Aptamers

VCAM‐1 is an important target in breast cancer treatment, and reducing its activity is important for both tumour prevention and therapy.^[149]^ Notably, VCAM‐1 aptamers have been used to image hormones and detect cerebral vascular inflammation in vivo.^[150]^

### 6.5 Aptamers for AMD

AMD is the most prevalent cause of blindness worldwide. Currently, one aptamer is in clinical use, whereas two others are still undergoing clinical trials.

#### 6.5.1 Macugen (Pegaptanib)

Macugen, a nucleic acid aptamer targeting VEGF165, is used in the eye to inhibit VEGF165 activity, providing therapeutic effects.^[151]^ It is the only aptamer currently approved for clinical use by the FDA.^[152]^

#### 6.5.2 E10030 (Fovista)

Also known as pegpleranib, this polyethylene glycol‐modified nucleic acid aptamer exhibits strong antitumour activity both in vitro and in vivo.^[153]^ Additionally, when used in combination with anti‐VEGF drugs, pegpleranib effectively promotes the regression of new blood vessels.^[154]^

#### 6.5.3 ARC1905 (Zimura)

ARC1905 is a complement inhibitor^[155]^ used for the treatment of both dry and wet forms of AMD.^[156]^

### 6.6 Aptamers Not Yet in Clinical Use

There are many aptamers that have not been used clinically, we present here NOX‐A12 and NOX‐H94.

NOX‐H94: The NOX‐H94 aptamer, lexaptepid pegol, is selective for target proteins in humans and crab‐eating monkeys, but is unable to recognise target proteins in rodents, and therefore cannot be used in the clinic under species‐restricted conditions.^[157]^

NOX‐A12: NOX‐A12 is an L‐type RNA oligonucleotide (Spiegelmer) that binds and neutralises CXCL12 and is used to interfere with CXCL12 and cell mobilisation in the tumour microenvironment. However, NOX‐A12 inhibits bone marrow haematopoiesis in myeloma.^[158]^

## 7 Challenges of Aptamer Therapy

**Figure 5:**
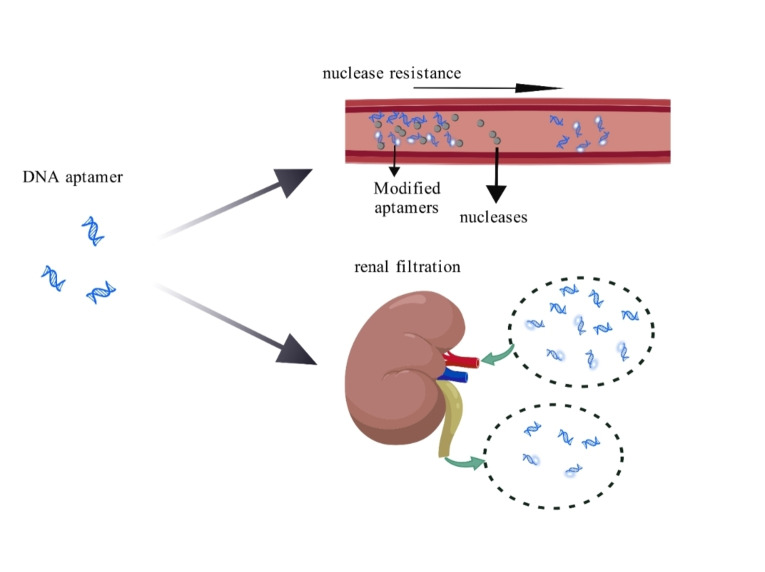
Aptamers face two major problems after entering the body: nuclease and renal filtration. (Created with BioGDP.com).

As a class of smaller nucleic acid sequences, aptamers have a high sensitivity to nucleases and low bioavailability (due to renal filtration) (Figure 5). These factors contribute to their limited in vivo application, posing one of the major challenges in aptamer development.^[159]^ To enhance their usability, aptamers are often modified by altering the backbone or side chains, adding non‐natural nucleotides, and capping their ends to prevent degradation by nucleases.^[51]^ These modifications also helped to further improve the success rate of the SELEX method.^[160]^

Nuclease resistance: Nucleases can cause rapid degradation of nucleic acids under many conditions, especially in medical settings. To obtain more stable nucleic acid molecules, chemical modifications have been introduced to enhance nuclease resistance. However, this method has potential drawbacks. Notably, the introduction of chemical groups may disrupt the affinity between the aptamer and its target.^[161]^ Moreover, introducing chemical groups into the nucleic acid backbone can significantly reduce nuclease degradation rates. For example, replacing the polar 2′‐OH group with 2′‐amino, 2′‐fluoro, or 2′‐O‐methyl modifications can enhance nuclease resistance,^[162]^ and thiophosphate esters can substitute for the phosphate diester bonds.^160^, ^163^

2′‐Amino pyrimidine: In modifications, 2′‐amino is commonly used to replace the nuclease‐sensitive 2′‐OH group. Lin et al. were the first to use a 2′‐amino‐containing library to screen aptamers for human neutrophil elastase.^[164]^ Although there are reports indicating that aptamers selected after 2′‐amino modification can be effective, these aptamers often do not function well during synthesis. Consequently, they are not used as therapeutic aptamers.^[165]^

2′‐Fluoro pyrimidine: Oligonucleotides containing 2′‐fluoro pyrimidine were used to produce Macugen (pegaptanib sodium), the first aptamer‐based drug approved by the FDA.^[166]^ Macugen^[152]^ is an anti‐vascular endothelial growth factor (VEGF) aptamer primarily used to treat age‐related macular degeneration (AMD) and ocular vascular diseases^[167]^

2′‐O‐methyl nucleotides: 2′‐O‐methylation is a common post‐transcriptional modification, with each ribosome containing >100 2′‐O‐methyl nucleotides.^[168]^ In 2006, the first siRNA with one 2′‐O‐methyl‐modified sense strand and one thiophosphate‐modified antisense strand was reported for the use of non‐natural drugs in oligonucleotide therapy. In 2005, the first fully modified aptamer, using 2′‐O‐methyl, was reported.^[169]^ A library containing these modified nucleotides was employed to screen four therapeutic aptamers. That same year, Burmeister et al. conducted SELEX on VEGF165 using an oligonucleotide library with varying levels of 2′‐O‐methyl content.^[170]^

Renal filtration: Chemical modifications significantly enhance aptamer resistance to nucleases. However, due to their small size, aptamers are sometimes cleared by the kidneys before they can perform their biological functions in vivo. The most effective approach is to conjugate aptamers to large nanomaterials such as polyethylene glycol (PEG). This increases the molecular weight of the aptamer–nanomaterial complex, surpassing the renal filtration threshold of 40 kDa.^[171]^ To date, PEGylated aptamers not only increase blood circulation time but also enhance nuclease resistance and reduce toxic accumulation in non‐target tissues.^[172]^ In addition to PEG, other nanomaterials can be used to modify aptamers and reduce their renal clearance. Aptamers conjugated with nanomaterials not only enhance nuclease resistance but also increase the affinity of the complex for the target.

In summary, aptamer nuclease resistance and in vivo usability can be improved by employing one or more modification strategies. However, no one‐size‐fits‐all modification approaches are available for specific aptamers. Some modification methods may affect the affinity between the target molecule and aptamer. Therefore, when designing modifications, it is important to tailor the approach based on aptamer structure and evaluate its effectiveness in achieving the desired outcomes.

## Summary and Outlook

This review introduces the various SELEX methods and nucleic acid aptamers for small molecules, highlighting that method selection depends on the target molecule. Each method has distinct advantages and limitations. Overall, nucleic acid aptamers are efficient, highly sensitive, and capable of rapid analysis. However, they still face the following issues: (1) Most aptamers are selected in vitro, and their binding effectiveness in vivo is not well‐established. (2) The structure of aptamers can change due to factors such as temperature, salinity, and pH, leading to instability or loss of specificity. In addition, aptamers are filtered by the kidneys and have a short circulation time in the body. (3) Biological samples contain proteins, nucleic acids, and small molecules such as lipids, carbohydrates, uric acids, and inorganic salts. These factors can affect the specific binding of aptamers to their targets, thereby affecting their specific recognition and application in clinical diagnostics.

Furthermore, the antibody market is gradually maturing, which may hinder aptamer development. Despite these challenges, aptamers hold significant promise, and it is anticipated that they will increasingly be used for clinical diagnosis and treatment.

## Conflict of Interests

The authors declare no conflict of interest.

## Biographical Information

*Song Liu received his Bachelor's degree from Shanxi Medical University in 2023, where he systematically mastered the core knowledge of medical theory. Subsequently, I went to Beijing Anzhen Hospital of Capital Medical University to pursue my master's degree in Pathology and Pathophysiology, during which I focused on cutting‐edge research on aptamers. Through continuous exploration and practice, I have deepened my understanding of the mechanisms of disease occurrence and development, and I am committed to applying aptamer technology to the diagnosis and treatment of diseases*.

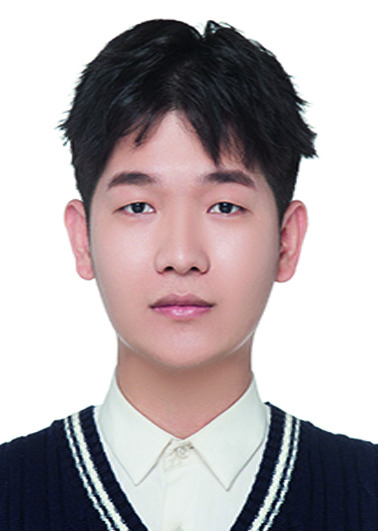

## Biographical Information

*Xiaolu Li graduated from North China University of Science and Technology with a Bachelor of Medicine in 2019. She then joined Capital Medical University to pursue her PhD in Pathology and Pathophysiology under the supervision of Dr Jiang, where she researched molecular epidemiology, cardiovascular disease metabolomics, and actively explored aptamer technology, working on new breakthroughs in medical diagnosis and treatment*.

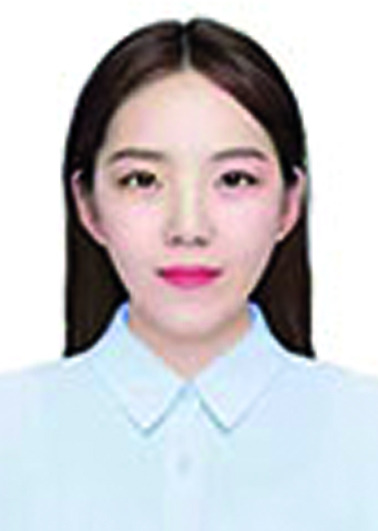

## Biographical Information

*Huyang Gao graduated from Shanxi Medical University in 2023, which gave him a solid medical foundation. He then enrolled in the Graduate School of Life Sciences of Guangxi Medical University to pursue a master's degree in regenerative medicine, focusing on the research of aptamers in the field of regenerative medicine. and intertwined with nanomaterials, he is committed to exploring the application of aptamers in promoting tissue repair and regeneration*.

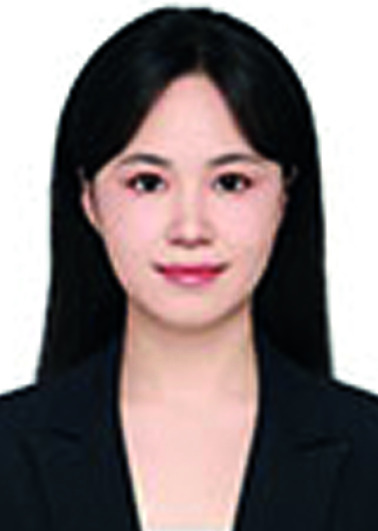

## Biographical Information

*Chen Jing received her Bachelor's degree from Chengde Medical University, China, in 2018. She pursued her Master's in Pathology & Pathophysiology at Capital Medical University, studying mechanisms of inflammation microenvironment imbalance harming cardiac structure and function. After obtaining her Master's in 2021, she joined Jiang Lab to explore the impact of small molecules, including aptamers, on heart diseases like atherosclerosis*.

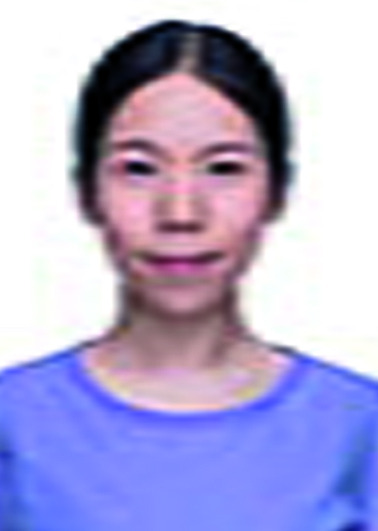

## Biographical Information

*Hongfeng Jiang, Ph.D, is a professor of pathophysiology at Capital Medical University. His work focuses on disease pathogenesis, translational study, and molecular diagnosis development of cardiovascular disorders, especially on identification of cardiovascular disease‐related proteins and intervention strategies of major chronic cardiovascular diseases. He has published over eighty peered reviewed research papers. He serves as an editorial board member of the Chinese Journal of Atherosclerosis, and Chineae Journal of CardiovascularMedicine. He is a member of the Metabolic Small Molecule Committee of the Chinese Society of Pathophysiology, and a member of the Lipids and Lipoprotein Committee of the Chinese Society of Biochemistry and Molecular Biology*.

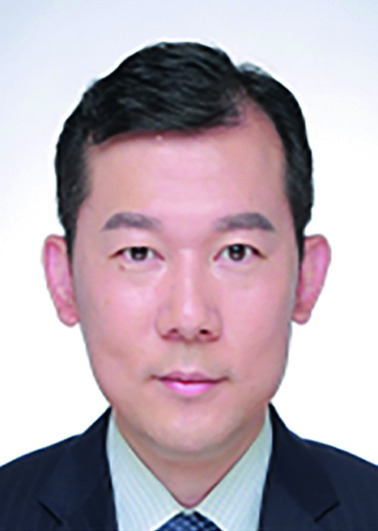

## Data Availability

The data that support the findings of this study are available on request from the corresponding author. The data are not publicly available due to privacy or ethical restrictions.
